# Bibliometric and Visual Analysis of Vascular Calcification Research

**DOI:** 10.3389/fphar.2021.690392

**Published:** 2021-07-15

**Authors:** Qian Dong, Qingchun Liang, Ying Chen, Jinhe Li, Lihe Lu, Xiongqing Huang, Qin Zhou

**Affiliations:** ^1^ Department of Anesthesiology, The First Affiliated Hospital of Sun Yat-sen University, Guangzhou, China; ^2^ Department of Anesthesiology, The Third Affiliated Hospital of Southern Medical University, Guangzhou, China; ^3^ Department of Pathophysiology, Zhongshan School of Medicine, Sun Yat-Sen University, Guangzhou, China

**Keywords:** vascular calcification1, chronic kidney disease2, atherosclerosis3, vascular smooth muscle cell4, citespace5, VOSviewer6, bibliometrics7

## Abstract

**Background:** Extensive studies related to vascular calcification (VC) were conducted in recent years. However, no bibliometric analysis has systematically investigated this topic. Our study aimed to determine the hotspots and frontiers of VC research in the past decade and provide a reference for future scientific research directions and decision-making in the VC field.

**Methods:** VC studies were acquired from the Web of Science Core Collection. Bibliometric and visual analyses were performed using CiteSpace, VOSviewer, and Microsoft Excel software.

**Results:** A total of 8,238 English articles on VC research published in 2011–2020 were obtained. In the past decade, annual publications and citations showed a significant growth trend, especially in 2018–2020. The most productive country, institution, journal and author are the United States, the University of California System, *PLOS ONE*, and Budoff MJ, respectively. The most frequently cited country, journal, and author are the United States, *Journal of the American College of Cardiology*, and Floege J, respectively. “Vascular calcification,” “atherosclerosis,” “chronic kidney disease,” and “cardiovascular disease” are the primary keywords. The burst keywords “revascularization,” “calciprotein particle,” “microRNA,” and “microcalcification” are speculated to be the research frontiers.

**Conclusion:** The main research hotspots in the VC field are the molecular mechanisms and prognosis of VC in patients with chronic kidney disease or cardiovascular disease. In addition, endovascular therapy and the development of new drugs targeting signal pathways for VC will become the focus of future research. Moreover, non-coding RNAs related to the diagnosis and treatment of VC are great research prospects.

## Introduction

Vascular calcification (VC) is defined as the abnormal deposition of calcium and phosphorus minerals in the arterial intima or media that can lead to reduced vascular wall compliance (Gourgas et al., 2018). VC is a common pathological manifestation of aging, atherosclerosis, hypertension, diabetes, and chronic kidney disease (CKD) (Poterucha and Goldhaber, 2016; Li et al., 2019). Among pathological conditions, CKD is most closely related to VC. Compared with the general population, CKD patients have a higher incidence and faster progression of VC (Shobeiri et al., 2010; Zhang et al., 2020). In addition, VC plays a key role in the pathogenesis of aortic valve disease, coronary artery disease and peripheral arterial disease (Park et al., 2021). Furthermore, its progression is highly related to increased cardiovascular morbidity and mortality (Shobeiri et al., 2010). Thus, we must pay closer attention to the occurrence and development of VC.

VC was previously thought to involve the passive deposition of calcium phosphate crystals within the vessel wall. However, since the 1990s, extensive studies have shown that VC is an active and regulated process similar to bone development (Shioi et al., 1995; Kong et al., 2018). Recent studies suggested that VC is a complicated biological process, depending on balance between calcification promoters and inhibitors (Lee et al., 2020). The calcification promoters include inflammation, oxidative stress, apoptosis, hyperparathyroidism and hyperphosphatemia (Yan et al., 2011; Song et al., 2017; Zhang et al., 2020). They can promote the differentiation of vascular smooth muscle cells (VSMCs) into osteogenic cells (Shao et al., 2010; Dong et al., 2020). In contrast, it is well-known that calcification inhibitors including PPi, MGP, fetuin-A, osteoprotegerin, osteopontin, Klotho, magnesium and SNF472 inhibit the differentiation of VSMCs into osteoblasts (Bäck et al., 2019; Perelló et al., 2020). VC occurs when the expression of calcification promoting factors is increased or the expression of calcification inhibitors is decreased. Increasing evidence has shown that the osteogenic differentiation of VSMCs is the core mechanism of VC. Multiple studies conducted in atherosclerosis or CKD animal models and human samples have suggested the existence of ossification/chondral metaplasia and the expression of osteoblasts in the pathological arterial wall (Rattazzi et al., 2005; Speer et al., 2009). However, the exact molecular mechanisms underlying VC remain unclear.

Continuous studies of VC mechanisms have made great contributions to the exploration of the best treatment to improve patient prognosis. However, no efficient therapy is approved for the treatment of VC until now, although various treatment strategies targeting pathological conditions (CKD, cardiovascular disease and osteoporosis) are available. These interventions include phosphate binders, sodium thiosulphate, calcimimetics, vitamin K, vitamin D, myoinositol, denosumab, and tissue-nonspecific alkaline phosphatase inhibitors (Singh et al., 2021). Several studies to date have demonstrated that the overexpression or inhibition of non-coding RNAs (ncRNAs) may provide promising avenues for VC therapy. Recently developed genome editing technologies, such as base editors and Cas13, can be used for VC treatment by targeting ncRNAs (Ryu et al., 2021). Additionally, emerging evidence has demonstrated that autophagy and lysosomal function are positive regulators of VC. Thus, the development of novel, highly selective autophagy or lysosome inducers may provide new therapeutic strategies (Neutel et al., 2020). In short, treatments based on molecular mechanisms, cellular signaling pathways, and gene regulation have extraordinary research value and therapeutic potential. However, an effective treatment strategy for VC has yet to be determined.

Several methods can be used to conduct a quantitative analysis of the literature, such as a traditional review, a main path analysis (Yu and Pan, 2021), and bibliometrics. Among them, bibliometrics is a comprehensive knowledge system that integrates mathematics, statistics, and philology and focuses on publication quantity. Bibliometrics has been widely employed to explore development trends and research frontiers in various research fields (Chen, 2006). CiteSpace software is a document visualization analysis software that was gradually developed under the background of bibliometrics and data visualization. It can display the basic knowledge and hotspots of a certain research field through visualization and predict evolution trends and research frontiers (Chen et al., 2012).

VOSviewer is a bibliometric software used to construct relationship networks and visualize data. Its outstanding feature is that it has strong graphics capabilities and is suitable for processing large-scale data (Perianes-Rodriguez et al., 2016). Using bibliometric methods, VC literatures can be visually analyzed, and the research results of the past decade can be displayed from multiple dimensions, laying the foundation for the in-depth development and research of VC worldwide. Although numerous studies have been conducted in the field of VC, no bibliometric research has examined VC to date. Therefore, we aimed to select the Web of Science Core Collection (WoSCC) database as a data source using CiteSpace, VOSviewer, and Microsoft Excel to display the knowledge base, developmental trends, and emerging hotspots of VC research.

## Materials and Methods

### Search Strategy

Relevant literatures were obtained from the SCI-E database of the WoSCC on February 1, 2021. The search formula was “TS = (vascular calcification OR arterial calcification OR aortic calcification OR vascular smooth muscle cell mineralization OR vascular smooth muscle cell calcification).” The search results were confined by language (English), literature type (article), and publication year (2011–2020). Ultimately, 8,238 records were identified.

### Data Analysis

CiteSpace 5.7. R4, VOSviewer 1.6.16, and Microsoft Excel 2019 were used to analyze and visualize the literature on VC research.

The visualization map created by CiteSpace consists of nodes and lines. The nodes in the graph represent the node types, such as country, institution, author, keyword, and reference. The size of each node stands for the frequency, and the connections between nodes represent cooperation, co-occurrence, or co-citation relationships. Node size is proportional to its frequency by type. The number of lines represents the degree of the connection between the nodes. Betweenness centrality is applied to quantify the importance of a node’s position in the network. The higher the betweenness centrality, the greater the number of connections in the network passing through the node. Nodes with a betweenness centrality greater than 0.1 are often marked with purple circles (Zheng and Wang, 2019). In our study, countries, institutions, and co-cited references were selected as nodes for the visualization analysis. Burst keywords were also detected using CiteSpace. The basic parameters were as follows: time slicing (2011–2020), years per slice (1), top N per slice (50), pruning (pathfinder and pruning sliced networks), and visualization (cluster view-static and show merged network).

VOSviewer can be used to construct a scientific knowledge network and display the structure, evolution, and cooperation of the research field (Van Eck and Waltman, 2010). In this study, VOSviewer was applied to visually analyze co-authorship, co-cited journals, and keyword co-occurrence as well as construct the density maps.

Microsoft Excel was used to construct the tables and demonstrate the annual national trends in publications and citations.

## Results

### Temporal Trends of Publications and Citations

A total of 8,238 English articles related to VC were included. There were 135,220 and 16.41 total and mean citations, respectively. The H-index count was 117. The H-index represents H papers published by the journal/author/country, each of which was cited at least H times. It can be used to evaluate the scientific impact of the journal, author, or country.

**FIGURE 1:**
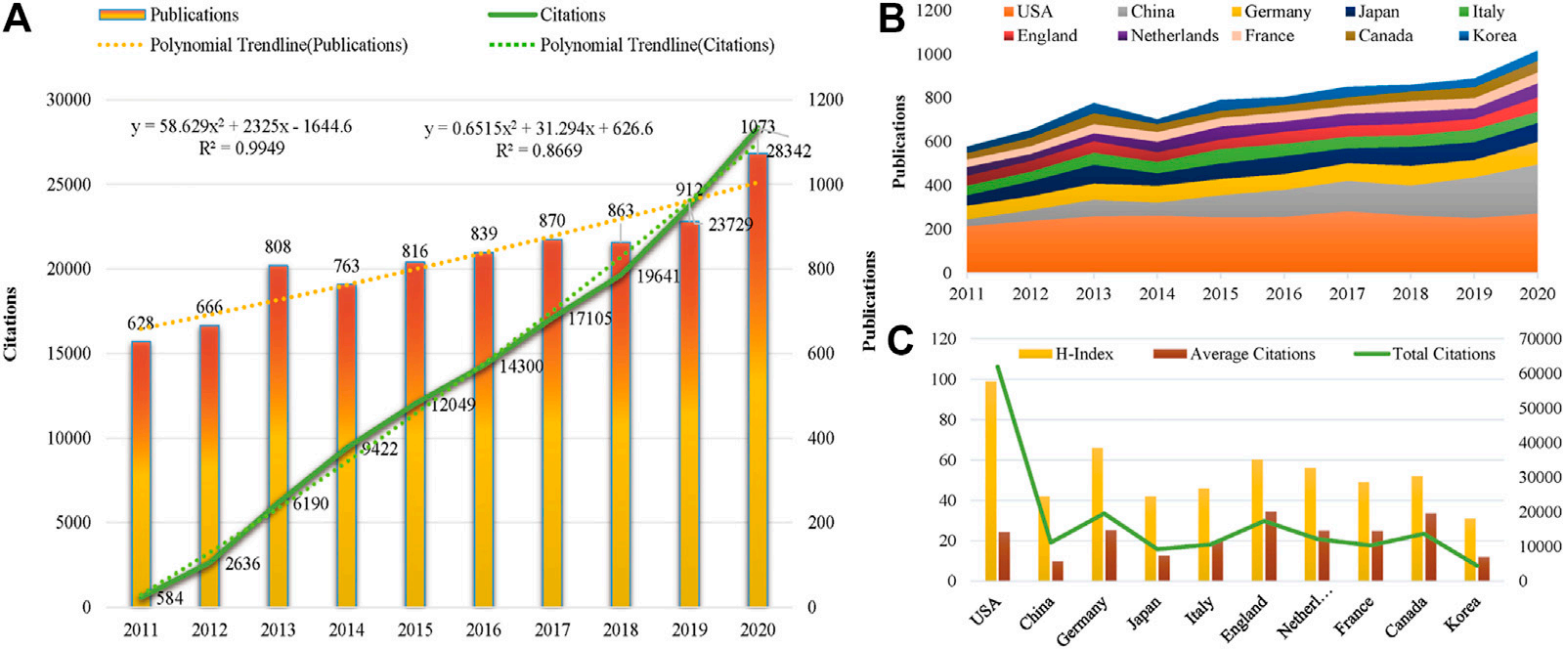
Trends in publications and citations of vascular calcification research. **(A)** The annual trends of global publications and citations. **(B)** The temporal trends of publications from the top 10 countries. **(C)** H-index, average citations (citations per article), and total citations of the top 10 countries.

From 2011 to 2020, the publications in the VC field generally showed an upward trend except for a slight decline in 2014 and 2018 (Figure 1A). A total of 1985 articles were published from 2019 to 2020, a high-yield and rapid growth stage in this field. As shown in Figure 1A, the annual number of citations were increased sharply in the past decade, especially from 2018 to 2020. The linear fitting of VC-related articles showed a significant correlation (R^2^ = 0.9949) between the year and the citations. The above results demonstrate that VC has received widespread attention from global scholars and that extensive research was conducted recently.

### Distribution of Countries and Institutions

**FIGURE 2:**
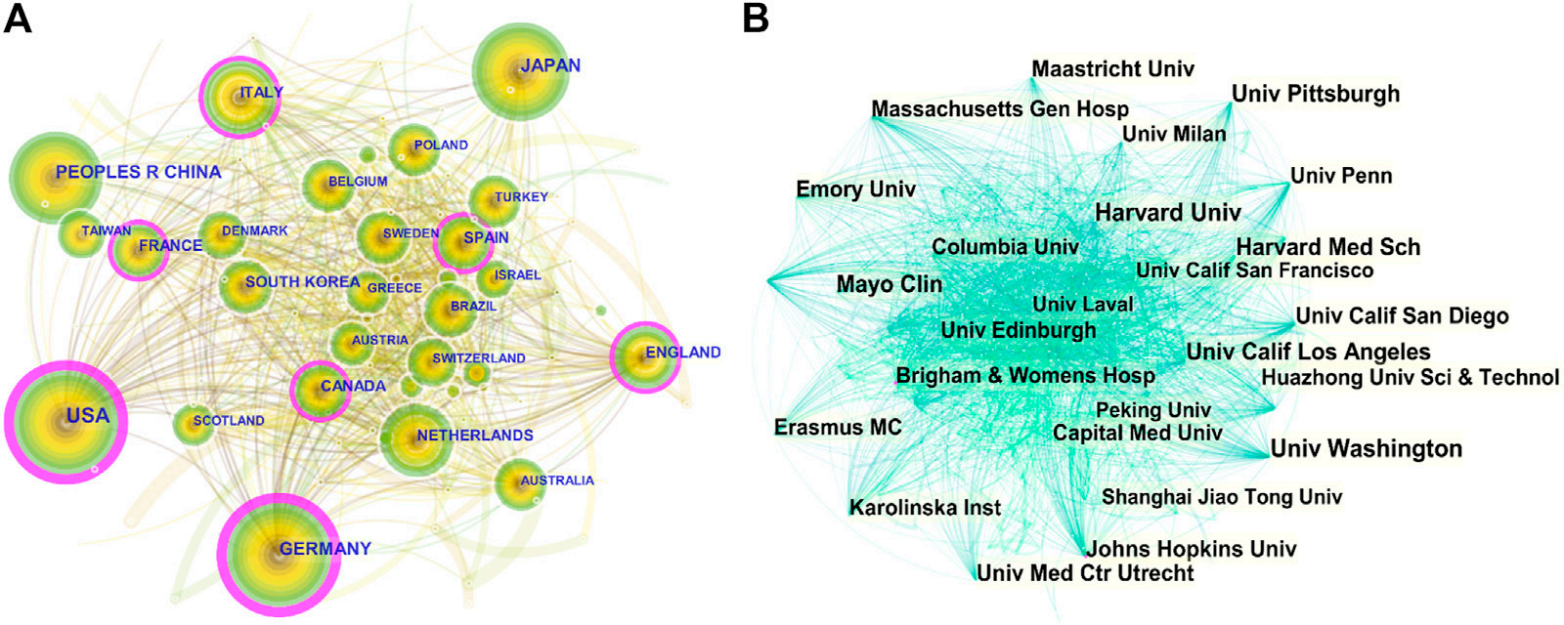
CiteSpace visualization map of countries and institutions involved in vascular calcification research. **(A)** Collaboration network of countries. *N* = 104, *E* = 798. *N* represents the number of network nodes. *E* represents the number of connections. **(B)** Collaboration network of institutions. *N* = 480, *E* = 3,382.

All literatures were distributed among 104 countries and 7,677 institutions. The United States had the most publications, with 2,555 (31.01%) articles, followed by China [1,128 (13.69%)], Germany [781 (9.48%)], Japan [729 (8.85%)], and Italy [536 (6.51%)] (Table 1). As shown in Figure 2A, the nodes of the United States, Germany, France, Canada, Spain, Italy, and England are marked with a purple circle, representing close cooperation with each other. The annual national publications (Figure 1B) and citations (Figure 1C) of the top 10 productive countries were further identified. The overall trend of annual publications by these countries exhibited a steady growth from 2011 to 2018 and a sharp increase beginning in 2019. In terms of mean citations, the top three countries were England (34.45), Canada (33.59), and Germany (32.57). The United States (99), Germany (66), and England (60) were the top three countries with a high H-index. The above results indicate that the United States, Germany, England, and Canada have great influence in the field of VC research.

**TABLE 1:** Top 10 countries by publications, citations and centrality.

Rank	Country	Publications	% of 8,238	Total Citations	Average Citations	H-index	Rank	Country	Centrality
1	United States	2,555	31.01	61,994	24.26	99	1	United States	0.28
2	China	1,128	13.69	11,146	9.88	42	2	Germany	0.24
3	Germany	781	9.48	19,570	25.06	66	3	France	0.15
4	Japan	729	8.85	9,205	12.63	42	4	Canada	0.15
5	Italy	536	6.51	10,597	19.77	46	5	Spain	0.14
6	England	504	6.12	17,361	34.45	60	6	Italy	0.12
7	Netherlands	489	5.94	12,171	24.89	56	7	England	0.12
8	France	416	5.05	10,314	24.79	49	8	Netherlands	0.06
9	Canada	408	4.95	13,706	33.59	52	9	Switzerland	0.06
10	Korea	373	4.53	4,450	11.93	31	10	Turkey	0.05

As shown in Table 2, the University of California System [367 publications (4.45%)], Harvard University [350 publications (4.25%)], and the National Institute of Health Care [246 publications (2.99%)] were the main research forces in this field. The centrality of Hopkins University and Brigham and Women’s Hospital was more than 0.1, indicating that these institutions were the key hubs for promoting international cooperation between relevant research institutions (Figure 2B). Among top ten institutions, most of them are American institutions (Table 2), suggesting that the United States is the leading driving force, and still dominates in this research field.

**TABLE 2:** Top 10 institutions distributed by publications and centrality.

Rank	Institution	Publications	Original Country	Institution	Centrality	Original Country
1	Univ Calif System	367	United States	Johns Hopkins univ	0.11	United States
2	Univ Harvard	350	United States	Brigham and Women’s Hosp	0.11	United States
3	INSERM	246	France	Univ Calif San Diego	0.08	United States
4	Univ Calif Los Angeles	189	United States	Karolinska inst	0.08	Sweden
5	Univ London	167	England	Emory univ	0.07	United States
6	Brigham and Women’s Hosp	155	United States	Univ Penn	0.06	United States
7	Johns Hopkins univ	154	United States	Univ Coll London	0.06	England
8	US department VA	153	United States	NSERM	0.06	France
9	Univ Washington	151	United States	Univ Leiden	0.06	Netherlands
10	Univ Seattle	149	United States	Univ Washington	0.05	United States

### Distribution of Authors and Co-Cited Authors

**FIGURE 3:**
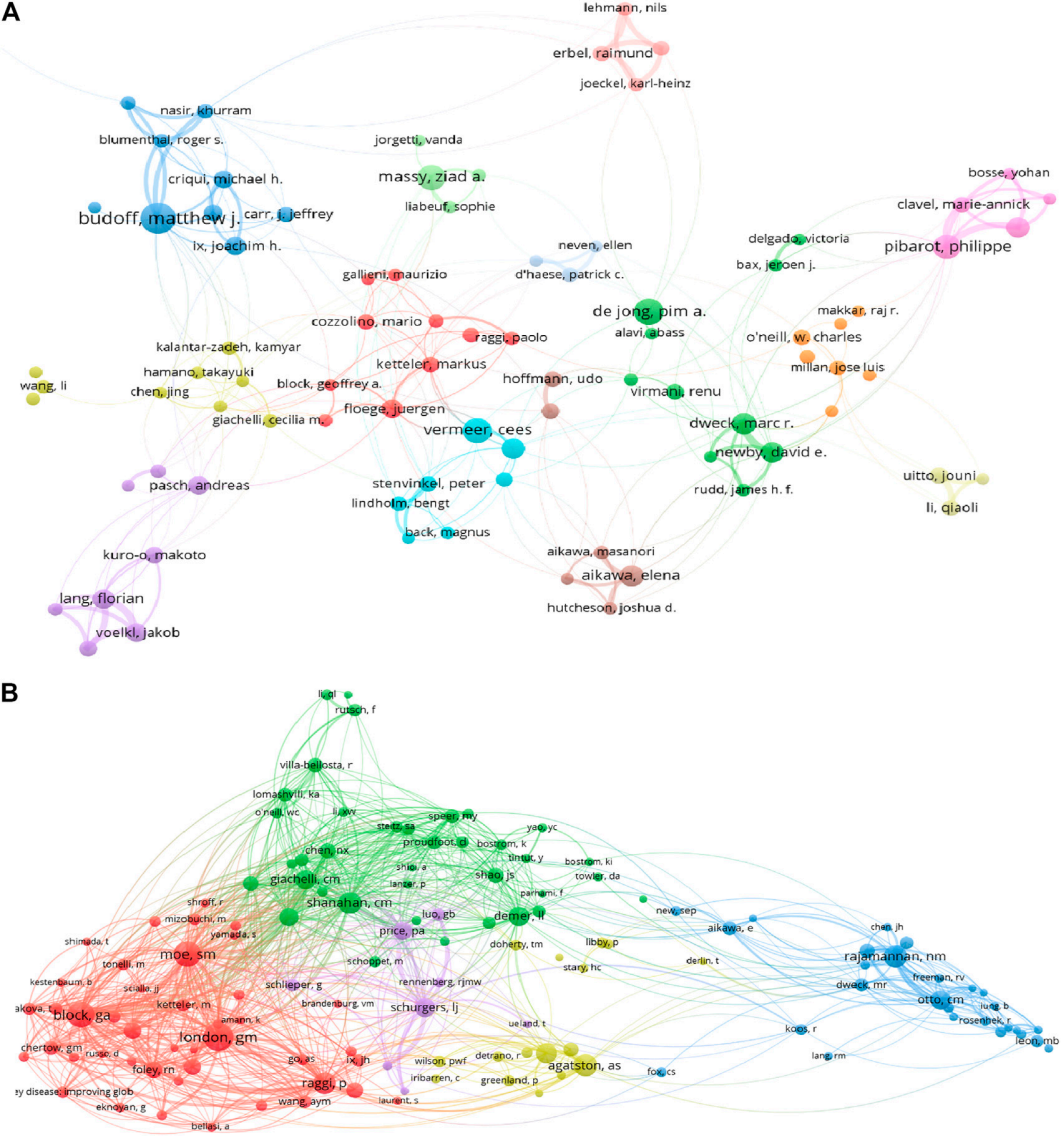
VOSviewer visualization map of authors and co-cited authors devoted to vascular calcification research. **(A)** Cooperation network of authors. Of the 42,046 authors, 113 had published at least 15 documents. **(B)** Co-citation network of authors. Of the 90,638 co-cited authors, 254 had at least 100 citations.

A total of 37,697 authors contributed to VC research. Budoff MJ, the most productive author from the United States, published 68 articles, followed by Wang Y (63), De Jone PA (54), and Massy ZA (53). The top three most published authors were Floege J (2,452), Pibarot P (2,302), and Budoff MJ (2,280). The above authors’ institution distribution showed that most institutions originated from North America and Europe (Table 3). As shown in Figure 3A, Budoff MJ cooperated closely with Criqui MH, Blumenthal RS, and Blaha MJ. In addition, Floege J collaborated frequently with Block GA, Raggi P, and Ketteler M. As shown in Figure 3B, London GM had the highest co-citations, followed by Block GA and Moe SM, and all of their co-citations exceeded 800. The above analysis suggests that they have a strong academic reputation in this area.

**TABLE 3:** Top 10 authors distributed by publications and citations.

Rank	Author	Publications	Country	Institution	Cited author	Total Citations	Average Citations	H-index	Country	Institution
1	Budoff MJ	68	United States	Univ Washington	Floege J	2,452	66.27	21	Germany	Rhein Westfal TH Aachen
2	Wang Y	63	China	Cent S univ	Pibarot P	2,302	50.04	25	Canada	Univ Laval
3	DE Jong PA	54	Netherlands	Uuniv med CTR utrecht	Budoff MJ	2,280	32.47	22	United States	Univ Washington
4	Massy ZA	53	France	Amiens univ Hosp	Dweck MR	2,151	52.46	21	Scotland	Univ Edinburgh
5	Vermeer C	49	Netherlands	Maastricht univ	Newby DE	2,056	55.57	20	Scotland	Univ Edinburgh
6	Pibarot P	46	Canada	Univ Laval	Vermeer C	1,996	39.92	27	Netherlands	Maastricht univ
7	Dweck MR	41	Scotland	Univ edinburgh	Schurgers LJ	1,817	49.11	22	Netherlands	Maastricht univ
8	Liu Y	39	China	Harbin med univ	Shanahan CM	1,798	74.92	19	England	Kjings Coll London
9	Wang L	38	China	Peking univ	Massy ZA	1,757	33.15	28	France	Amiens univ Hosp
10	Floege J	37	Germany	Rhein westfal TH Aachen	Aakawa E	1,621	45.03	23	United States	Harvard med Sch

### Distribution of Journals

**FIGURE 4:**
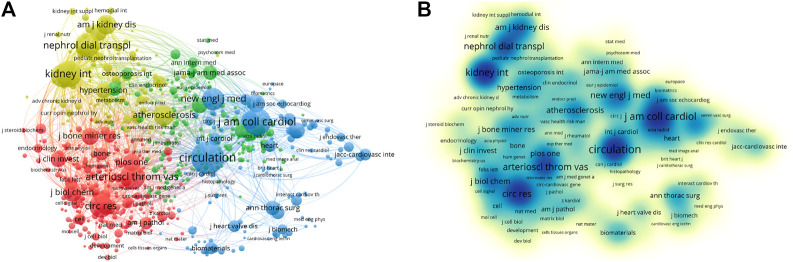
VOSviewer visualization map of most commonly cited journals related to vascular calcification research. **(A)** Co-citation network of journals. Of the 12,149 most commonly cited journals, 99 had published at least 500 studies. **(B)** The density map of the most commonly cited journals. The dark blue nodes represent journals with large numbers of citations.

All articles were published in 1,476 journals, including 19 with more than 50 articles. The impact factor (IF) and journal quartile were obtained from Journal Citation Reports 2019. The top three prolific journals were *PLOS ONE* (IF2.74), *Atherosclerosis* (IF3.919), and *Arteriosclerosis Thrombosis and Vascular Biology* (IF6.604). In addition, the *Journal of the American College of Cardiology* (IF20.589) had the greatest number of citations, followed by *Arteriosclerosis Thrombosis and Vascular Biology* (IF6.604) and *PLOS ONE* (IF2.74) (Table 4). The top three co-cited journals were *Circulation* (IF23.609), *Journal of the American College of Cardiology* (IF20.589), and *Kidney International* (IF8.52) (Figures 4A,B). Most journals listed in Table 4 were classified in Q1 or Q2, and these results indicate that the above journals have strong academic performance in the field of VC research.

**TABLE 4:** Top 10 journals distributed by publications and citations.

Rank	Journal	Publications	% of 8,238	IF(JCR 2019)	JIF quartile	Journal	Total Citations	Average Citations	H-index	IF(JCR 2019)	JCR quartile
1	Plos one	283	3.44	2.74	Q2	J Am Coll Cardiol	6,907	71.21	45	20.589	Q1
2	Atherosclerosis	196	2.38	3.919	Q2	Arterioscl throm Vas	5,423	35.68	41	6.604	Q1
3	Arterioscl Throm Vas	152	1.85	6.604	Q1	Plos one	4,699	16.6	36	2.74	Q2
4	Nephrol Dial transpl	126	1.53	4.531	Q1	Nephrol Dial transpl	3,746	29.04	35	4.531	Q1
5	Sci Rep-UK	111	1.35	3.998	Q1	Circ res	3,641	98.41	28	14.467	Q1
6	Catheter Cardio inte	100	1.21	2.044	Q2	Kidney int	3,446	54.7	33	8.945	Q1
7	Int J Cardiol	92	1.12	3.229	Q2	New engl J med	3,355	671	5	74.699	Q1
8	BMC Nephrol	78	0.95	1.913	Q3	Atherosclerosis	3,308	16.88	32	3.919	Q2
9	J Am Coll Cardiol	77	0.93	4.605	Q1	Circulation	3,039	70.67	28	23.603	Q1
10	Am J Cardiol	75	0.91	2.57	Q2	J Am Soc Nephrol	2,748	80.82	25	9.274	Q1

### Analysis of Highly Cited Articles and Co-Cited References

As shown in Table 5, the top three cited articles were as follows. 1) “Two-year outcomes after transcatheter or surgical aortic-valve replacement” by Kodali SK in 2012 (1,573 citations). This study conducted a 2-years follow-up of patients with aortic valve stenosis or calcification in the PARTNER trial. These results support transcatheter aortic valve replacement as an appropriate alternative to surgical treatment for high-risk patients, but paravalvular regurgitation occurred more frequently after transcatheter aortic valve replacement (Kodali et al., 2012). 2) “Mechanisms of plaque formation and rupture” by Bentzon JF in 2014 (664 citations). This article discussed the mechanism of the initiation and development of atherosclerotic plaques, the sudden deposition of life-threatening thrombi, and the concepts of plaque burden, mobility, and vulnerability (Bentzon et al., 2014). 3) “Klotho deficiency causes vascular calcification in chronic kidney disease” by Hu MC in 2011 (551 citations). The study reported that Klotho improved vascular calcification by enhancing the excretion of phosphate in the urine, maintaining glomerular filtration, and directly inhibiting the absorption of phosphate by vascular smooth muscle. Therefore, a Klotho deficiency can lead to the development of VC (Hu et al., 2011). Notably, the number of citations does not fully reflect the quality of the article, because the citations of articles published earlier are generally higher than those of the latest articles. We have to consider the influence of time as a confounding factor on citation analysis.

**TABLE 5:** Top 10 cited literatures.

Rank	Citations	Author	Title	Source	Year	Column	Page	DOI
1	1,573	Kodali SK	Two-year outcomes after transcatheter or surgical aortic-valve replacement	New engl J med	2012	366	1,686	10.1056/NEJMoa1200384
2	664	Bentzon JF	Mechanisms of plaque formation and rupture	Circ res	2014	114	1852	10.1161/CIRCRESAHA.114.302721
3	551	Hu MC	Klotho deficiency Causes Vascular Calcification in chronic kidney disease	J Am Coll Cardiol	2011	22	124	10.1681/ASN.2009121311
4	542	Chertow GM	Effect of Cinacalcet on cardiovascular disease in patients undergoing dialysis	New engl J med	2012	367	2,482	10.1056/NEJMoa1205624
5	493	Joshi NV	F-18-fluoride positron emission tomography for identification of ruptured and high-risk coronary atherosclerotic plaques: a Prospective clinical trial	Lancet	2014	383	705	10.1016/S0140-6736(13)61754-7
6	474	Shanahan CM	Arterial calcification in chronic kidney disease: Key roles for calcium and phosphate	Circ res	2011	109	697	10.1161/CIRCRESAHA.110.234914
7	434	Thanassoulis G	Genetic associations with Valvular Calcification and Aortic Stenosis	New engl J med	2013	368	503	10.1056/NEJMoa1109034
8	328	Block GA	Effects of phosphate binders in moderate CKD	J Am Soc Nephrol	2012	23	1,407	10.1681/ASN.2012030223
9	325	Raggi P	The ADVANCE study: a randomized study to evaluate the effects of cinacalcet plus low-dose vitamin D on vascular calcification in patients on hemodialysis	Nephrol Dial transpl	2011	26	1,327	10.1093/ndt/gfq725
10	323	Hayashida K	Transfemoral aortic valve implantation new criteria to predict vascular complications	JACC-cardiovasc inte	2011	4	851	10.1016/j.jcin 2011.03.019

**FIGURE 5:**
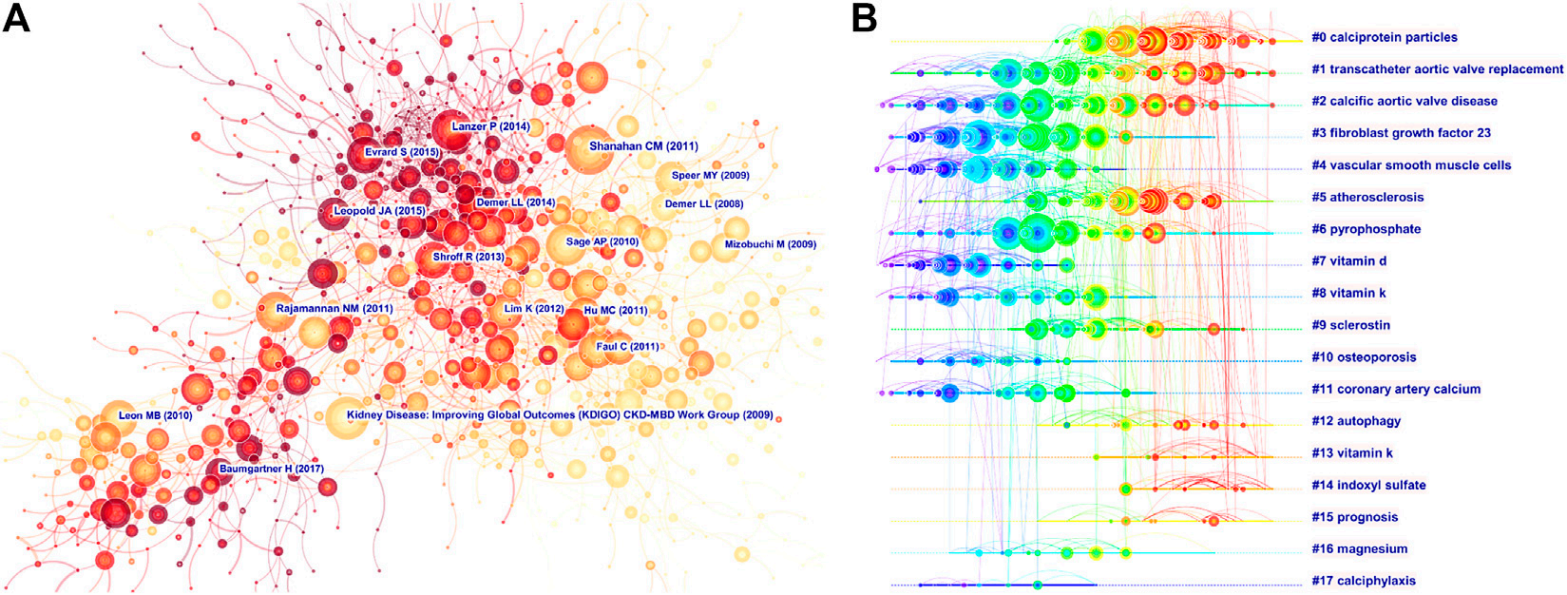
Analysis of most commonly cited references related to vascular calcification research. **(A)** Co-citation network of references. **(B)** Timeline view of most commonly co-cited references.

As shown in Figure 5A, “Shanahan CM (2011),” “Lanzer P (2014),” “Sage AP (2010),” and CKD-MBD Work Group (2009)” were frequently co-cited references. A cluster analysis showed that the high-frequency terms related to VC research were mainly clustered into 18 categories (Figure 5B). The top five clusters were “calciprotein particles,” “transcatheter aortic valve replacement,” “calcified aortic valve disease,” and “fibroblast growth factor 23,” “vascular smooth muscle cells” and “atherosclerosis” (Table 6). The silhouette (S) value refers to the average contour value of the cluster. It is generally considered that, with an s > 0.5, the cluster is reasonable, while with an s > 0.7, the cluster is convincing (Chen et al., 2012). In our study, all of the silhouette values of the top 10 clusters were above 0.7, which indicated that the clusters were convincing. “calciprotein particles” (Cluster ID 0#) was the largest cluster with 125 references, and literatures related to calciprotein particles could be currently primary hotspots.

**TABLE 6:** Top 10 largest clusters of co-cited references.

Cluster ID	Top terms	Size	S-value	Mean (year)
#0	Calciprotein particles	125	0.829	2016
#1	Transcatheter aortic valve replacement	116	0.977	2013
#2	Calcific aortic valve disease	103	0.933	2011
#3	Fibroblast growth factor 23	96	0.908	2010
#4	Vascular smooth muscle cells	81	0.886	2009
#5	Atherosclerosis	75	0.903	2013
#6	Pyrophosphate	64	0.897	2011
#7	Vitamin d	63	0.918	2007
#8	Vitamin k	58	0.919	2009
#9	Sclerostin	44	0.849	2013

### Analysis of Keywords

**FIGURE 6:**
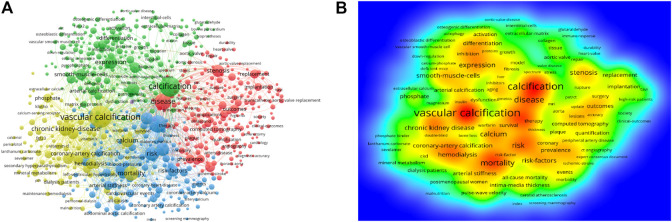
Analysis of all keywords in studies related to vascular calcification research. **(A)** VOSviewer visualization map of co-occurring keywords. Of the 18,983 keywords, 133 had at least 100 co-occurrences. **(B)** The density map of keywords. The closer the keyword node color is to red, the higher the frequency of its co-occurrence.

As shown in Table 7, the keywords used at high frequency were: “vascular calcification,” “calcification,” “atherosclerosis,” “chronic kidney disease,” and “mortality”. Keywords with high centrality were: “vascular calcification,” “chronic kidney disease,” “computed tomography,” “cardiovascular disease,” and “atherosclerosis”. As shown in Figure 6A, the yellow cluster was constituted mainly of “vascular calcification,” “chronic kidney disease,” “calcium,” “coronary artery calcification,” and “mortality”. The cluster mainly explored the mechanisms and pathogenesis of VC in patients with CKD or on dialysis. The blue cluster focused on “atherosclerosis,” “cardiovascular disease,” “risk-factors,” “progression,” “arterial stiffness” and “cardiovascular events”. It primarily probed various pathophysiological changes associated with cardiovascular disease, including atherosclerosis, arterial calcification, and arterial stiffness. Furthermore, the association between VC progression and the incidence of cardiovascular adverse events was also investigated. In addition, “stenosis,” “computed tomography,” “replacement,” “implantation” and “outcomes” were chief components of the red cluster. This study compared the therapeutic effects of open valve replacement and transcatheter valve replacement for aortic valve or heart valve stenosis. Moreover, the green cluster primarily consisted of “calcification,” “inflammation,” “expression,” “mechanisms” and “smooth muscle cell”. In this cluster, a series of *in vitro* experiments was performed to describe the molecular mechanism of VC. According to the density map of keywords, “vascular calcification,” “chronic kidney disease,” “hemodialysis,” “coronary artery calcification,” “mortality,” “vascular smooth muscle cells,” and “expression” had higher weight (Figure 6B).

**TABLE 7:** Top 10 keywords by frequency and centrality.

Rank	Frequency	Keyword	Centrality	Keyword
1	2,170	Vascular calcification	0.54	Vascular calcification
2	2,117	Calcification	0.31	Chronic kidney disease
3	1,437	Atherosclerosis	0.28	Computed tomography
4	1,240	Chronic kidney disease	0.22	Cardiovascular disease
5	981	Mortality	0.18	Atherosclerosis
6	928	Cardiovascular disease	0.18	Stenosis
7	715	Calcium	0.17	Calcification
8	710	Expression	0.15	Expression
9	676	Inflammation	0.13	Smooth muscle cell
10	632	Stenosis	0.12	Stage renal disease

Burst keywords can reflect emerging academic trends and new topics, predict the Frontier research direction, and explore potential hotspots in a field. As shown in Table 8, the keywords were closely concentrated between 2017 and 2020. Furthermore, the burst keywords in this period mainly included “revascularization,” “microRNA,” “microcalcification,” “matrix vesicle” and “calciprotein particles”. Burst detection can be divided into two aspects: the first is closely related to revascularization of coronary atherosclerosis and heart valve stenosis, while the second chiefly focused on the molecular mechanism and relevant treatment of VC.

**TABLE 8:** Top 8 keywords with strongest citation burst.

Keyword	Strength	Begin	End	2011–2020
Atherosclerosis mesa	6.93	2014	2017	▂▂▂▃▃▃▃▂▂▂
Paravalvular regurgitation	9.87	2016	2018	▂▂▂▂▂▃▃▃▂▂
TNF-α	6.79	2016	2017	▂▂▂▂▂▃▃▂▂▂
Revascularization	8.9	2017	2020	▂▂▂▂▂▂▃▃▃▃
Microrna	7.45	2017	2020	▂▂▂▂▂▂▃▃▃▃
Microcalcification	7.41	2017	2020	▂▂▂▂▂▂▃▃▃▃
Matrix vesicle	7.41	2017	2020	▂▂▂▂▂▂▃▃▃▃
Calciprotein particle	6.76	2018	2020	▂▂▂▂▂▂▂▃▃▃

## Discussion

### General Information

In the present study, we utilized bibliometric technology to analyze literature related to VC. Our results indicated that the annual publications and citations of VC have shown a significant growth trend in recent years. As the main driving force, the United States had the highest number of publications and total citations. Furthermore, eight of the top 10 prolific institutions were from the United States, such as the University of California System and Harvard University. In addition, close cooperation was observed between American institutions, which maximized its geographical advantages and further strengthened its academic influence on VC research. Although England ranked sixth in the number of publications, it had the highest mean number of citations, suggesting very high quality of its publications. Moreover, the University of London in England had a high number of publications and collaborated closely with other institutions. The above results showed that European and North American institutions were dominant in the VC research direction, whereas China had no leading institutions in this field.

Additionally, our results demonstrated that Budoff MJ was the most productive author with a high number of citations. He mainly focused on imaging and made significant contributions to the identification of VC lesions. He also participated in various sub-studies of multi-ethnic research on atherosclerosis. These studies mainly focused on the impact of valve calcification and coronary atherosclerosis on the incidence and mortality of patients with CVD (Budoff et al., 2011). He also explored the association between VC progression and patient prognosis (Fashanu et al., 2020). We also found that the most commonly cited author was Floege J from Germany. He mainly studied the occurrence and development of VC in patients with CKD under certain pathological conditions. These pathological states include secondary hyperparathyroidism, hypermagnesemia, anticoagulant drugs, and vitamin K deficiency (Floege, 2015; Floege, 2018). Moreover, the author with the highest number of co-citations was London GM, who mainly studied arterial aging, stiffness changes, and vascular remodeling in CKD patients (London et al., 2016; London, 2018). The above authors have high academic reputations in VC research and have contributed significantly to developments and advancements in this field.

Journal Citation Reports 2019 was used to obtain the impact factor (IF) and quartile (Q) of a journal category (Chen L. et al., 2020). The Journal Citation Reports division divides the journals in the same discipline into four equal parts, with the top 25% being Q1 and 25–50% being Q2. We found that most of the productive journals were classified as Q1 or Q2, and *Arteriosclerosis Thrombosis* and *Vascular Biology* (IF6.604, Q1) had the highest IF. Although *PLOS ONE* (IF2.74, Q2) and *Atherosclerosis* (IF3.919, Q2) were the top two productive journals, their IF values were less than 5. This indicates that improving research quality while increasing output may contribute to enhancing their academic influence. Among the most commonly cited journals, the *New England Journal of Medicine* (IF74.699, Q1), *Circulation* (IF23.603, Q1), and *Journal of the American College of Cardiology* (IF20.589, Q1) had IF values higher than 20. This suggests that the three aforementioned journals published high-quality research with convincing and mature outcomes.

### Hotspots and Frontiers

Through the potent combination of top keywords and literature, we attribute the research hotspots as follows: 1) The mechanism of VC in CKD or dialysis patients, including inflammation (Yuan et al., 2020), VSMC phenotype of osteogenic differentiation (Yang et al., 2020), overexpression of calcification-promoting factors (oxidative stress) (Watanabe et al., 2020), parathyroid hormone (Imafuku et al., 2020), fibroblast growth factor 23 (Jiang et al., 2020) as well as the suppression of calcification inhibitors (Liu et al., 2018); 2) various pathophysiological changes (atherosclerosis, arterial calcification, arterial stiffness, etc.) accompanied by CVD, and the impact of these changes on the prognosis of patients (Stabley and Towler, 2017; Mszar et al., 2020); 3) comparison of therapeutic effects between open valve replacement surgery and minimally invasive transcatheter valve replacement for aortic valve or heart valve stenosis (Ielasi et al., 2021); and 4) molecular and cellular mechanisms of VC, including various molecular signaling pathways (Chen W.-J. et al., 2020) and abnormal proliferation (Miyata et al., 2020) or apoptosis (Boraldi et al., 2021) of cells that regulate VC.

Based on the analysis of burst keywords, we speculated that endovascular intervention measures for VC will become an emerging academic trend in VC research. These measures included minimally invasive transcatheter valve replacement for aortic valve or heart valve stenosis (Ielasi et al., 2021), percutaneous coronary intervention for coronary artery calcification (Jia et al., 2020), and intravascular lithotripsy (Dini et al., 2019) for peripheral arterial disease. Concurrently, signal transduction pathways have been found to play a significant role in VC occurrence and progression. Therefore, research on functional pathways has always been favored by researchers and will never become outdated. Recent popular pathways include the miR-126-3p-DKK1/LRP6 pathway (Zeng et al., 2021), NR4A1/DNA-PKcs/p53 pathway (Zhu et al., 2020), AMPK/OPA1 pathway (Chen W. R. et al., 2020), and Keap1/Nrf2 pathway (Cui et al., 2020). Extensive Researches on signal transduction pathways could provide novel strategies for the development of new drugs targeting VC. The field of ncRNAs has recently emerged as a focus of VC research and has received widespread attention from scholars worldwide. Accumulating evidence demonstrates that ncRNAs, including circular RNAs (Ryu et al., 2020), microRNAs (Chao et al., 2020), and long ncRNAs (Ryu et al., 2021), can modulate VC by acting as promoters or inhibitors and may play critical role in the prevention, diagnosis, and treatment.

### Limitations

To date, this study is the first bibliometric analysis of hotspots and dynamic frontiers of VC research in the past decade. In addition, three tools were applied to perform the survey simultaneously, enabling our research results to be more accurate and objective. However, the present study has some limitations. First, all data were extracted only from the WoSCC, and we did not search more databases, such as Google Scholar or PubMed. However, the WoSCC is an authoritative, comprehensive, and multidisciplinary core journal citation index database. Second, only English articles were included, which may have decreased the number of retrieved articles. And finally, the amount of literature related to VC is huge. With the rapid updating of hot topics and research frontiers of VC, we may have missed some research hotspots.

## Conclusion

Extensive studies related to VC have been conducted in the past decade, and the number of retrieved articles demonstrated an obvious growth trend, especially from 2019 to 2020. The most productive country, institution, journal, and author are the United States, the University of California System, *PLOS ONE*, and Budoff MJ, respectively. The most commonly cited country, journal, and author are the United States, *Journal of the American College of Cardiology*, and Floege J, respectively. The document with the highest number of citations is “Two-year outcomes after transcatheter or surgical aortic valve replacement” by Kodali SK in 2012. The main research foci in the VC field are the molecular mechanism of VC and the prognosis of VC in patients with CKD or cardiovascular disease. In addition, endovascular therapy and the development of new drugs targeting signal pathways for VC will become the foci of future research. Moreover, ncRNAs related to the diagnosis and treatment of VC have great research potential. In general, this study systematically analyzed the literatures on VC and reported the research results of the past decade in multiple dimensions, which can lay the foundation for the in-depth development and research of VC worldwide. Our results revealed the authors and institutions that can cooperate and provide reference for future research directions and scientific decision-making.

## Data Availability

The original contributions presented in the study are included in the article/Supplementary Material, further inquiries can be directed to the corresponding authors.
